# Endogenous Opioids in Wound-Site Neutrophils of Sternotomy Patients

**DOI:** 10.1371/journal.pone.0047569

**Published:** 2012-10-31

**Authors:** Hamdy Awad, Motaz Abas, Haytham Elgharably, Ravi Tripathi, Tykie Theofilos, Sujatha Bhandary, Chittoor Sai-Sudhakar, Chandan K. Sen, Sashwati Roy

**Affiliations:** 1 Comprehensive Wound Center and Davis Heart and Lung Research Institute, Department of Anesthesiology, Wexner Medical Center at the Ohio State University, Columbus, Ohio, United States of America; 2 Comprehensive Wound Center and Davis Heart and Lung Research Institute, Department of Surgery, Wexner Medical Center at the Ohio State University, Columbus, Ohio, United States of America; University of North Dakota, United States of America

## Abstract

**Background:**

Postoperative pain management is a critical aspect of patient care. The inflammatory state of the post-sternotomy surgical wound sensitizes nerve endings, causing pain. Unrelieved or improperly managed pain compromises wound healing. Peripheral opioid receptors play a major role in analgesia, particularly under inflammatory conditions where both opioid receptor expression and efficacy are increased. Leukocytic opioid peptides include β-endorphin (END), met-enkephalin (ENK), and dynorphin-A (DYN), with END and ENK being predominant.

**Methodology/Principal Findings:**

This work represents the first study of inflammatory cells collected from post-sternotomy wounds of patients undergoing cardiac surgery including coronary artery bypass grafting (CABG). Wound fluid (WF) and cells were collected from sternal wounds using a JP Blake drain at 24, 48, and 72 hours post sternum closure. Anti-CD15 staining and flow cytometry revealed that polymorphonuclear neutrophils (PMN) are the predominant cells present in wound fluid collected post-surgery. Compared to peripheral blood (PB) derived PMN, significant increases in CD177+/CD66b+ PMN were observed suggesting activation of wound-site PMN. Such activation was associated with higher levels of opioid peptide expression in PMN derived from WF. Indeed, increased level of opioid peptides in sternal wound environment was noted 72 h post-surgery. We demonstrate that WF contains factors that can significantly induce POMC transcription in human PMNs. IL-10 and IL-4 were abundant in WF and both cytokines significantly induced POMC gene expression suggesting that WF factors such as IL-10 and IL-4 contribute towards increased opioid peptide expression in wound-site PMN.

**Conclusions/Significance:**

This approach provided a unique opportunity to study the cross-talk between inflammation and opioid peptides in PMN at a sternotomy wound-site. Wound-site PMN exhibited induction of END and ENK. In addition, sternal wound fluid significantly induced END expression in PMN. Taken together, these data constitute first clinical evidence that human wound-site PMNs are direct contributors of opioids at the sternal wound-site.

## Introduction

An estimated 50–75% of surgical patients suffer from inadequate pain relief [1]. Pain, following cardiac surgery, may occur at the incision and sternotomy sites, chest tube sites, and vascular harvesting sites [2]. Inadequate postoperative pain control has been associated with compromised outcomes including poor wound healing [1].

Conventional opioids are powerful to alleviate severe pain, but their benefit is offset by potential side effects such as depression of breathing, nausea, clouding of consciousness, constipation, addiction, and tolerance [3]. The endogenous opioid peptide system represents one of the best-characterized clinically relevant endogenous analgesics mediators [4], [5]. Opioid receptors are widely expressed in the central and peripheral nervous system as well as in numerous non-neuronal tissues [6]. Findings from studies of both animal models as well as patients support the involvement of peripheral opioid receptors in analgesia, particularly under inflammatory conditions where both opioid receptor expression and efficacy are increased [5], [6]. The opioid peptides expressed in leukocytes include β-endorphin (END), met-enkephalin (ENK), and dynorphin-A, with END and ENK being predominant [6], [7], [8]. Polymorphonuclear neutrophils (PMN) express full and truncated mRNA for opioid peptide precursors [6].

During the last fifty years, fifty-nine analgesic drugs have been introduced to the market. Yet much of the critical needs of clinical pain management remains unmet [9]. The significance of immune cell-released peptides in the inflammatory milieu of human wounds is poorly understood. We developed an opportunity to study acute inflammatory responses in patients undergoing primary, elective, coronary artery bypass grafting (CABG) or other procedures involving sternotomy while they are hospitalized. This work provides patient-based evidence demonstrating the presence of opioid peptides and their precursor in the wound-site PMN.

## Results

**Figure 1:**
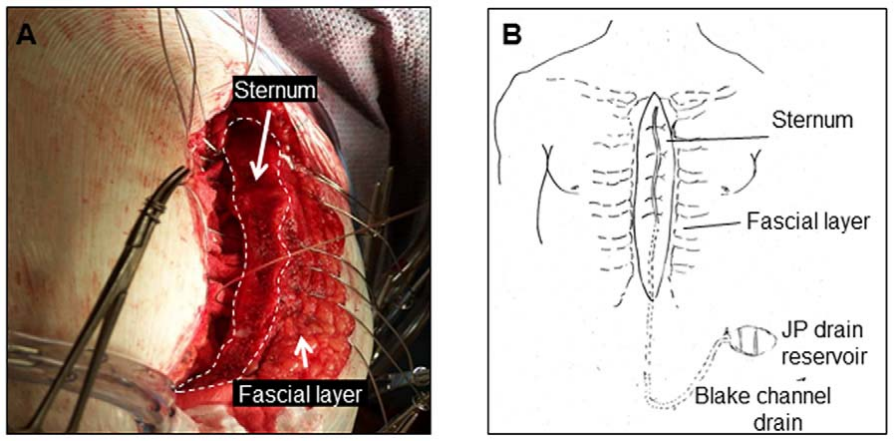
Fluid and cell collection from sternal wound environment. **A**, image of the sternal wound before closure where the Blake drain is yet to be placed; **B**, after closure of the sternum, the surgeon placed a Blake drain over the sternum, and then closed the wound in layers. The Blake drain was connected to a heparinized J-VAC bulb suction reservoir for wound fluid collection.

**Figure 2:**
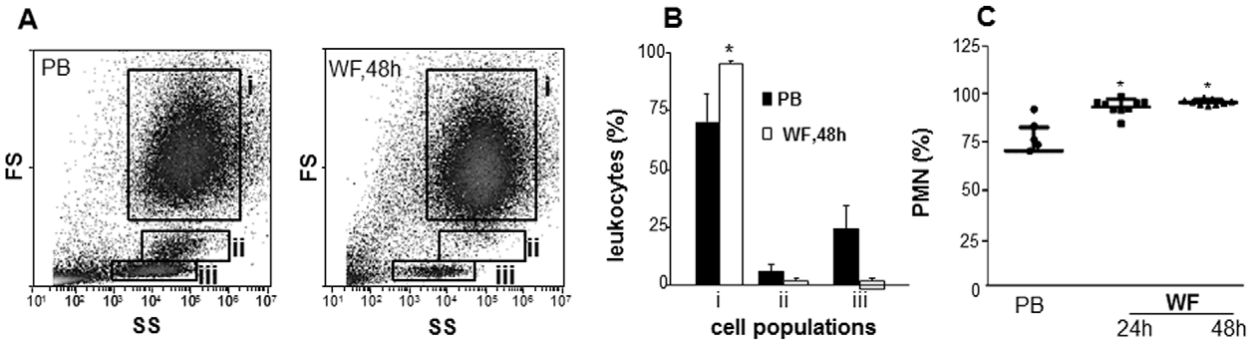
Cells in sternotomy wound-fluid are predominantly PMN. Wound cells isolated from WF were subjected to flow cytometry analysis. **A**, forward and sideward scatter (FS/SS) and flow cytometry analysis to sub-fraction wound and blood leukocytes. Based on FS/SS characteristics of blood leukocytes (PB, before surgery), three major cell populations were gated: ***gate i***, granulocytes; ***gate ii***, monocytes; ***gate iii***, lymphocytes; **B**, quantification of cells in each of the three gates (i–iii). Solid bar, blood at baseline; open bars, WF cells at 48 h post-surgery. Data are mean ±SD, n = 4. **C**, Neutrophil count expressed in % of total leukocytes as determined by microscopic differential count. Data presented as mean± SD, n = 7. *p<0.05 compared to PB.

This work developed a novel approach to isolate and study functionally intact cells and fluid from the sternal wound environment of sternotomy patients (Fig. 1). In flowcytometry, in addition to fluorescence, two types of light scatter are measured. The forward scatter (FS) is roughly proportional to the diameter of the cell, while the side scatter (SS) is proportional to the internal complexity such as granularity. For example the neutrophil granulocytes have higher side scatter than do lymphocytes, which are agranular. We used the FS/SS in flow cytometry analysis to sub-fraction the WF and PB-derived leukocytes. Based on classical FS/SS (size/granularity) characteristics of blood leukocytes (before surgery), three major cell populations were gated: *gate i*, granulocytes; *gate ii*, monocytes; and *gate iii,* lymphocytes (Fig. 2A). Using these gates, the % of major leukocyte populations in cells derived from WF was determined (Fig. 2A). Granulocytes were identified as the predominant cell type (over 90%) present in WF at 48 h post-surgery (Fig. 2B). Data obtained from FS/SS flow cytometry analysis were confirmed using clinical complete blood count (CBC) enumerating differential count in PB and WF samples (Fig. 2C).

**Figure 3:**
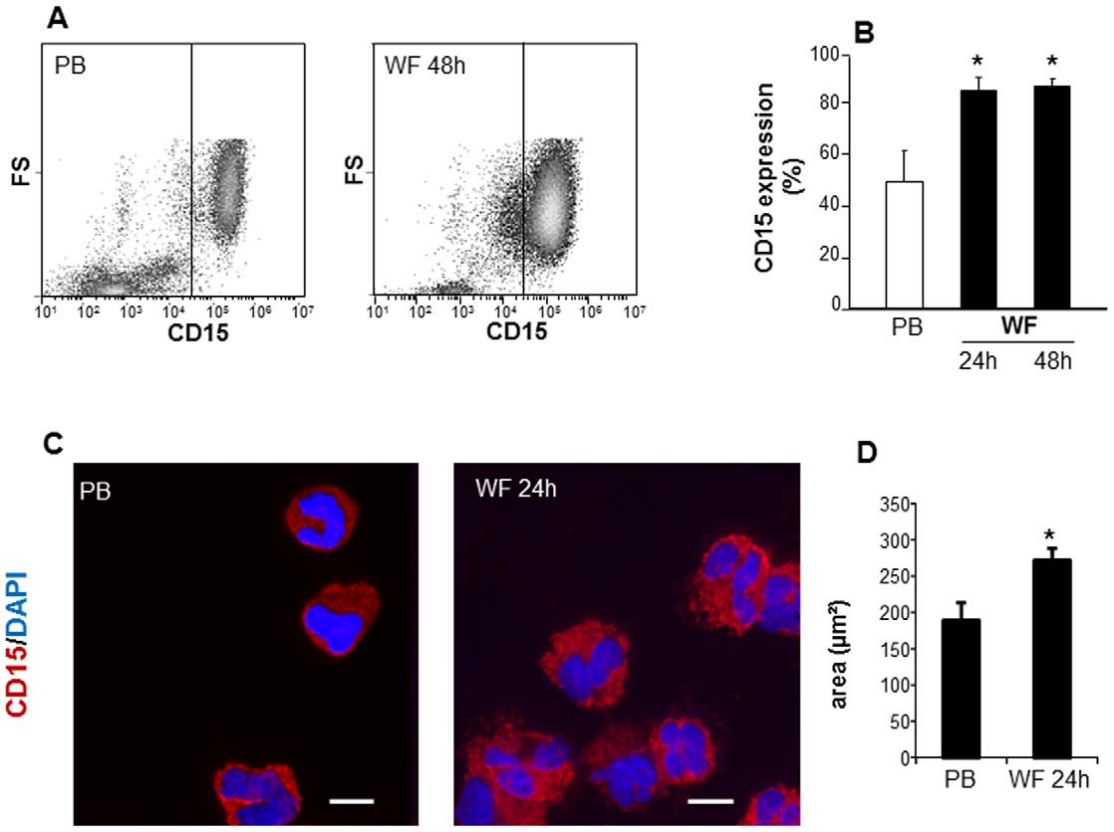
PMN in wound fluid are CD15 positive. PB and WF leukocytes were isolated and stained with FITC-conjugated anti-human CD15 followed by **A–B**, flow cytometry analysis or **C–D**, cytospin. **A**, representative flowcytometry plots showing increased CD15 positive cells in WF at 48 h post wounding as compared to peripheral blood at baseline (PB). **B**, CD15 expression analysis using flow cytometry. Data presented as mean±SD, n = 4. *p<0.05 compared to PB. **C**, Representative images of cytospun cells derived from WF or PB. Cells were immunostained with CD15 (red) and DAPI (nucleus, blue). Scale bar = 10 µm; **D**, size analysis of cytospun cells derived from WF and PB. Data are mean ± SD; *p*<0.05 compared to PB.

CD15 is a commonly used granulocyte marker [10]. PB or WF leukocytes were stained with CD15 antibody and subjected to flow cytometry analysis (Fig. 3A). The line drawn in the dot plot (FS vs CD15) separates CD15 negative (isotype control stained) and positive cells (Fig. 3A). Consistent with the FS/SS analysis, over 90% of WF derived cells were CD15 positive following 24 h or 48 h post-surgery as compared to PB at baseline (before surgery) where the fraction of CD15 positive cells was ∼50–60% (Fig. 3B). These data indicate that cells present in WF in the early inflammatory phase are predominantly CD15 positive PMNs. To morphologically characterize these cells, samples were mounted on a cytospin centrifuge and transferred onto slides followed by staining using the FITC-conjugated anti-CD15 antibody and examined under fluorescence microscope (Fig. 3C). Microscopic examination showed that CD15 positive PMN were the predominant cell types in wound fluid after 24 h or 48 h post-surgery. Size analyses using the area measurement tool in Axiovision (Zeiss) showed that the PMN derived from WF were significantly larger in area compared to the size of PMN derived from PB (Fig. 3D).

**Figure 4:**
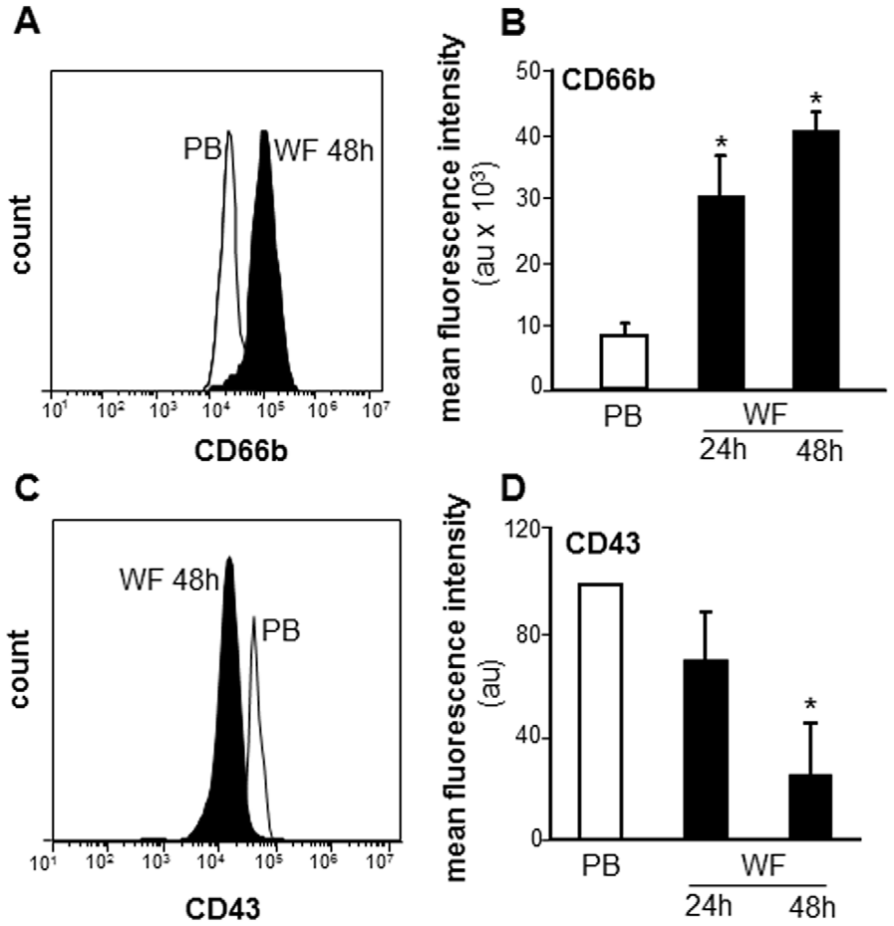
Cells from WF show activated PMN phenotype: increased CD66b and decreased CD43 expression. The WF and PB cells were subjected to flow cytometry analysis following immunostaining with CD66b or CD43. **A&C,** representative flowcytometry histograms of CD66b and CD43; **B&D,** quantification of CD66b and CD43 flow cytometry analysis. Data presented as mean±SD, n = 4. *p<0.05 compared to PB.

**Figure 5:**
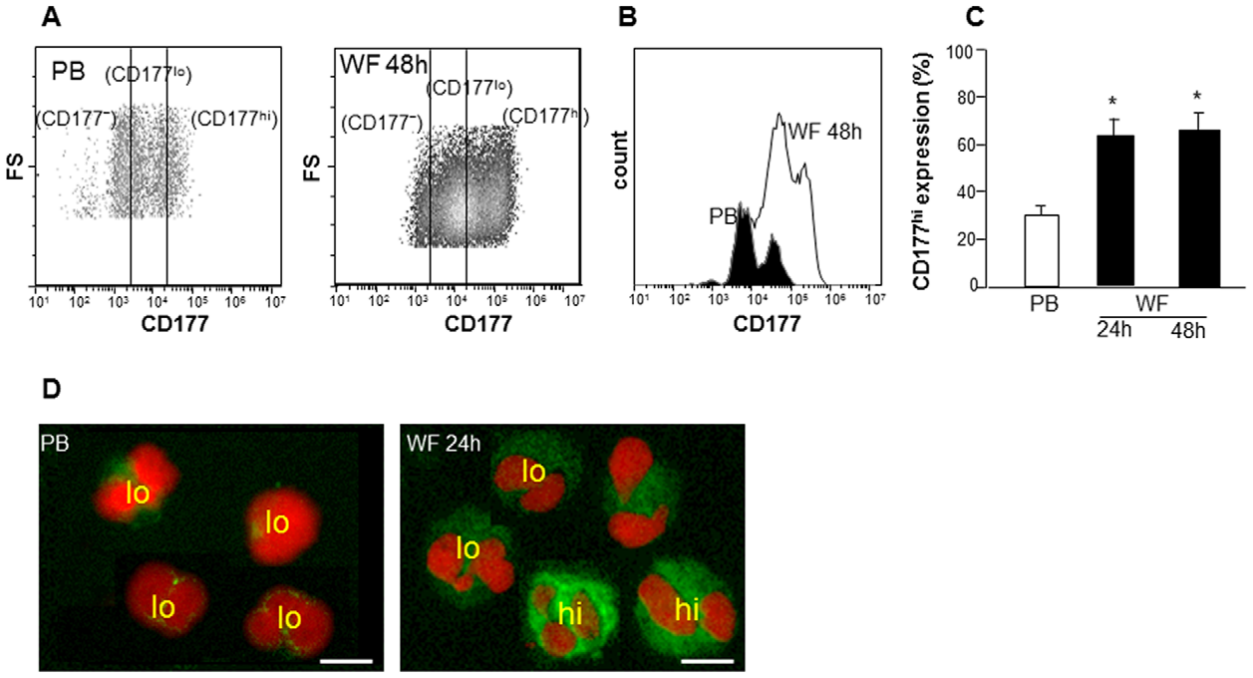
Increased CD177 expression in PMN derived from WF. Cells from WF and PB were immunostained with CD177 followed by flow cytometry or cytospin analysis. Representative **A**, scatter plot and **B**, histogram and **C**, quantification from flow cytometry analysis showing cells with low (lo) or high (hi) expression of CD177. **D**, Representative images of cytospun cells derived from WF or PB. The cells were immunostained with CD177 (green) and DAPI (nucleus, red). Scale bar = 10 µm.

Increased CD66b expression is involved with neutrophil activation and migration [11], [12]. CD43, a negatively charged membrane sialoglycoprotein leukosialin, is shed during neutrophil activation [13]. To determine the activation state of PMN at the sternal wound site, CD66b and CD43 surface expression were determined using immunostaining and flow cytometry showing mean fluorescence intensities (MFI). The expression of Cd66b was significantly up-regulated in WF whereas CD43 was significantly down-regulated at 48 h post-surgery (Fig. 4A–B). CD177 (HNA-2a, NB-1), expressed on PMN, interacts with PECAM-1 to facilitate neutrophil transmigration [11], [14]. Flow cytometry analysis revealed two distinct populations of PMN expressing either CD177 at low (CD177^lo^) or high levels (CD177^hi^; Fig. 5A). Fluorescence microscopy exhibited data consistent with that of flow cytometry (Fig. 5D). There was a significant increase already at 24 h post-surgery in the percentage of WF PMN with CD177^hi^ expression as compared to PB derived PMN (Fig. 5B–C). No further change in percent of CD177^hi^ positive PMN was observed. These data suggest that there is either an increase in migration of PMN subsets expressing high levels of CD177 at sternal wound site or an increase in the expression of this CD177 on PMN by factors present in wound environment.

**Figure 6:**
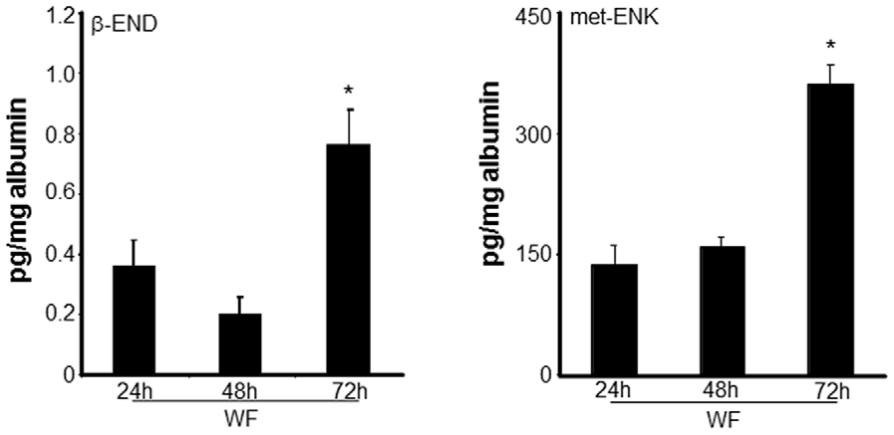
Increased endogenous opioid peptide levels in wound fluid at late inflammatory phase. Wound fluid (WF) was obtained from sternal wounds. Opioid peptide β-endorphin (END) and met-enkephalin (ENK) levels in WF was determined using ELISA at the indicated time points after surgery. The peptide levels were normalized to total albumin level in the fluid. Data are mean±SD (n = 5).* *p*<0.05 compared to 24 h post-surgery.

**Figure 7:**
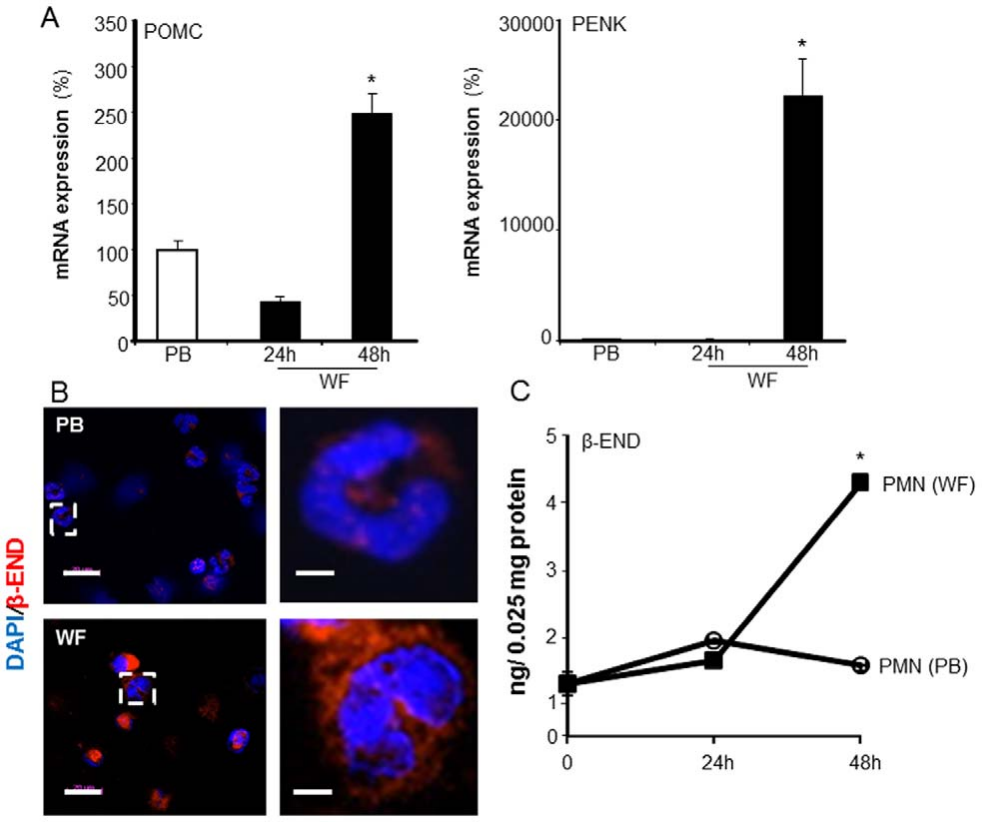
Increased endogenous opioid peptide expression in wound-site PMN. WF derived cells were isolated from sternal wounds. **A**, mRNA expression of pro-opiomelanocortin (POMC) or preproenkephalin (PENK), the precursors of END and ENK, respectively, were determined in WF and peripheral blood (PB) PMN using quantitative PCR (qPCR). Data are mean±SD (n = 3).* *p*<0.05 compared to PB; **B**, ***Left***: Representative images of cytospun cells derived from WF or PB. Cells were immunostained with anti-β-Endorphin (β-END; red) and DAPI (nucleus, blue). Scale bar = 20 µm. ***Right:*** zoomed images of white box shown in corresponding left panels. Scale bar = 5 µm. **C**, Intracellular opioid peptide β-endorphin (END) and met-enkephalin (ENK) levels in WF derived PMN determined using ELISA at the indicated time points after surgery. The peptide levels were normalized to total cellar protein levels. Data are mean±SD (n = 3).* *p*<0.05 compared to 24 h post-surgery.

Inflammatory mediators released from leukocytes are known to contribute to the generation of pain. Conversely, leukocytes produce opioid peptides that can effectively counteract pain [7]. Opioid peptides expressed in leukocytes include β-endorphin (END), met-enkephalin (ENK), and dynorphin-A (DYN) [15]. Using ELISA, the levels of the END and ENK were determined in WF. A significant increase in the levels of END and ENK was noted in WF at 72 h post-surgery (Fig. 6). The level of END and ENK in PB plasma was multifold higher as compared to the levels in WF at all the time points (data not shown). In PB, the release of END and ENK predominantly occur from pituitary gland and the brain [16], [17], [18]. To test whether increased END and ENK levels in WF were caused by increased expression of END and ENK in wound PMN, we examined both the transcripts (POMC and PENK) as well as actual intracellular levels of the opioid peptides in the PMN derived from WF. Compared to PB-derived PMN, a significant increase in both mRNA expression of precursor POMC and PENK mRNA (Fig. 7A) as well as the intracellular levels of END and ENK (Fig. 7B–C) was noted in PMN 48 h post-surgery.

**Figure 8:**
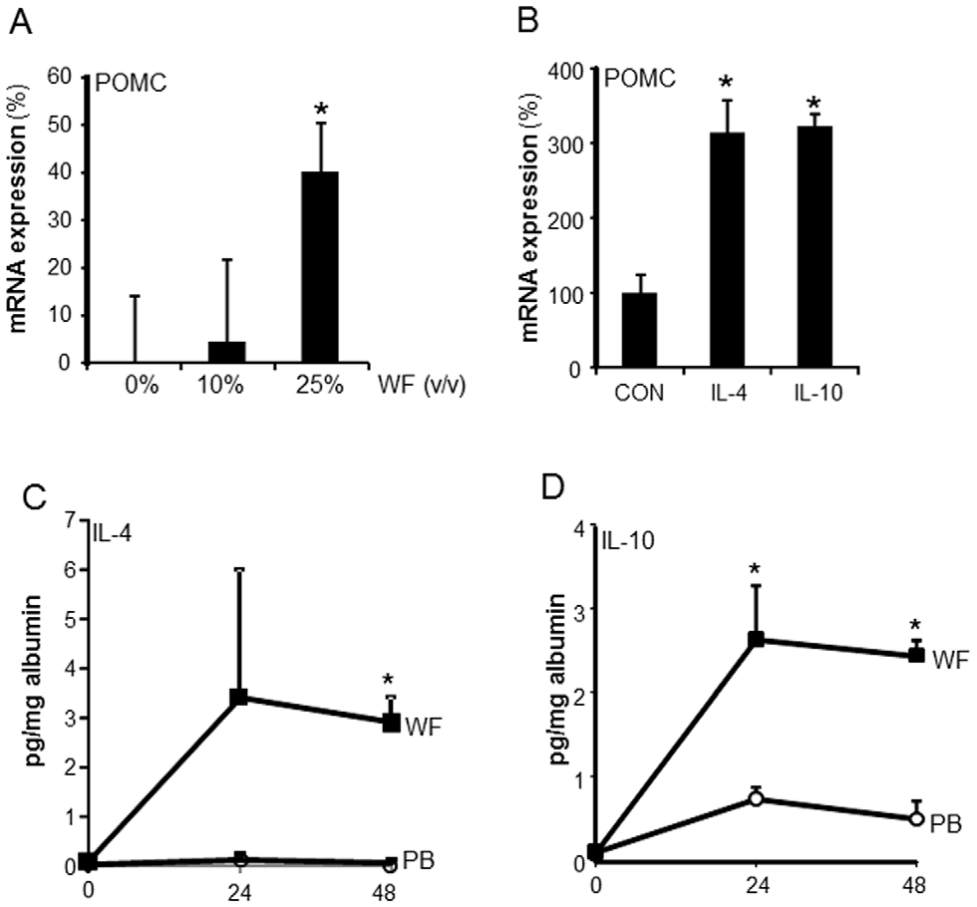
Sternal wound environment induces opioid peptide expression in PMN: presence of opioid inducing IL-4 and IL-10 in sternal wound environment. **A,** HL-60 cells were differentiated to PMN and then treated with varying dilutions (0–25%, v/v, 24 h) of wound fluid (WF) derived from sternal wounds. WF was sterile filtered and added directly to the PMN culture medium. POMC mRNA levels in WF treated PMN was measured using qPCR. Data are mean ± SD (n = 3).*, p<0.05 compared to 0% treated cells. **B**, HL-60 cells were differentiated to PMN and then were treated with 100 ng/ml of IL-10 or IL-4 for 48 h. The expression of POMC transcripts were measured using qPCR. Data are mean ± SD (n = 3). * p<0.05 compared to control. Both IL-4 and IL-10 that are abundantly present in WF significantly induced POMC mRNA expression. **C**, Levels of IL-4 and IL-10 in wound fluid (WF) and plasma were determined using ELISA at the indicated time points after surgery. The cytokine levels were normalized to the total albumin level in the fluid or plasma. Data are mean ± SD (n = 5). * *p*<0.01 compared to plasma.

Finally, we used cultured PMN to determine if wound environment is capable of inducing opioid peptide expression in PMN. Differentiation of the acute myeloid leukemia (AML) cell line HL-60 to mature PMN was induced by all trans-retinoic acid treatment. The differentiated cells were treated with varying concentration (0–25% v/v) of sternal WF for 24 h. Following 24 h, the expression of END precursor mRNA POMC was determined. WF (25% v/v) significantly induced expression of POMC expression in HL-60 derived PMN (Fig. 8A). POMC mRNA expression in PMN was also induced following treatment of the cells with IL-10 and IL-4 (Fig. 8B). Both of these cytokines were abundantly present in wound environment (Fig. 8C) suggesting that such factors present in wound environment may induce opioid peptide expression in wound site PMN.

## Discussion

Wound healing is a physiological response to injury that is conserved across tissue systems. The inflammatory stage is one of the early events following injury in which PMN, soon followed by macrophages, migrate into the wound site [19]. In this work we report that 24–48 h after cardiac surgery involving sternotomy, PMN represented the predominant cell population in WF. This is consistent with the literature reporting that in hip drain fluid collected 24 h after surgery 96±3% of the leukocytes in the WF were neutrophils [20]. We observed that WF PMN were significantly larger compared to the size of blood plasma PMN. The size of PMN has been suggested as an additional physical characteristic demonstrating heterogeneity among these cells [21]. Circulating human PB PMN have been shown to represent a continuum of sizes that demonstrates a positive correlation with the oxidative metabolism of the cells [21]. Whether the increased size of wound-site PMN was because of increased oxidative metabolism remains to be investigated.

CD66b, a member of the carcinoembryonic antigen adhesion molecule family, is an established marker of neutrophil activation [12], [22]. CD66b expression has been reported in PMN from inflamed synovial tissue (ST) compared to those from non-inflamed ST. CD66b is directly implicated in adhesion events during inflammation [23]. We report that wound-site PMN featured elevated expression of CD66b which was associated with lowered CD43 expression. CD43 or leukosialin is a sialic acid-rich molecule expressed in PMN [13]. Activation of PMN with classical activators such as phorbol myristate acetate (PMA) or by N-formyl-L-methionyl-L-leucyl-L-phenylalanine (FMLP) is known to decrease CD43 expression up to 80% [24]. Intriguingly, in our study, WF PMN showed elevated CD177 expression, an important marker of myeloproliferative diseases. In 1990, neutrophil specific antigen HNA-2a was found to be located on the 58–64 kD NB1 glycoprotein [25]. Later in 2001, the gene encoding HNA-2a and the NB1glycoprotein, CD177, were identified by Kissel and colleagues [26]. CD177 is implicated in transfusion-related acute lung injury [27], and is also induced in a number of inflammatory settings [14]. Taken together, induced CD66b, CD177 and repressed CD43 expression on WF PMN point towards activated phenotype of PMN in the sternal wound environment. An understanding of whether these cells at the wound site reflect recruitment of specific subsets of peripheral blood PMN, or activation of indiscriminately recruited cells at the sternal wound *milieu,* warrant further investigation.

Peripheral endogenous mechanisms of opioid analgesia are known to have direct clinical implications [7], [28]. Both animal and human clinical data support the involvement of peripheral opioid receptors in analgesia, particularly under inflammatory conditions [5], [6]. In patients undergoing knee surgery, blocking intra-articular opioid receptors by the local administration of naloxone resulted in significantly increased postoperative pain [5]. Peripheral opioid receptor-mediated analgesia has been evident in experimental human pain models. Administration of the peripherally restricted opioid agonist morphine-6-glucoronide (M6G) reduced hyperalgesia induced by freeze lesions and excessive muscle contraction [29].

Opioid peptides mediate interactions between the nervous system and the immune system [30], and are known to exist in specific leukocyte subpopulations at the wound site [31], [32]. These peptides are encoded by three different genes, pro-opiomelanocortin (POMC), prodynorphin and preproenkephalin (PENK). The precursor of END, POMC mRNA was originally reported in the bovine pituitary [33]. POMC mRNA is suppressed in mature, non-stimulated leukocytes. However, it is inducible under pathological conditions [34]. PENK mRNA, and the appropriate enzymes for posttranslational processing of PENK to ENK have also been detected in human monocytes [35]. This study provided first evidence demonstrating increased expression of END and ENK precursor mRNA as well as the peptide levels in human wound-site PMN. Previously, murine neutrophils and monocytes/macrophages invading the peritoneal cavity have been shown to express PENK mRNA in zymosan- or thioglycollate-induced experimental peritoneal inflammation. The wound environment was enriched with IL-10 and IL-4, cytokines known to be a potent inducer of opioid peptides [30] suggesting induced causative link between these inducers and opioid peptide expression in wound-site PMN. In the current study, the presence of END and ENK in the WF warrants further investigation of their source. The presence of precursor transcripts and opioids in wound-site PMN points towards these cells as a potential source of opioid peptides in the wound microenvironment.

This work reports a novel translational approach to temporally investigate inflammatory cell biology at an acute sternotomy wound site. This approach provides a unique opportunity to study the cross-talk between inflammation and opioid peptides in PMN at a sternal wound-site. Wound-site leukocytes collected from sternotomy patients showed an activated phenotype and contained high levels of both transcripts as well as the peptides of endogenous opioids. The wound fluid surrounding the wound tissue reflects the wound microenvironment and shapes the functional response of wound-related cells [36], [37]. This study provides direct evidence in humans, that the local wound environment may induce opioid peptide expression in wound–site PMN. Such opioids are released in the wound environment by the infiltrating inflammatory cells and may contribute toward peripheral analgesia at the wound-site.

## Materials and Methods

### Human subjects and sample collection

Subjects participating in the study were scheduled to undergo primary, elective, coronary artery bypass grafting (CABG) or other procedures involving sternotomy at The Ohio State University Wexner Medical Center (Fig. 1). Fourteen patients participated in this study, and demographic characteristics of these patients are presented in (Table 1). Protocols were approved by The Ohio State University's Institutional Review Board. Declaration of Helsinki protocols was followed and patients gave their written, informed consent. After closure of the sternum, the surgeon placed a Blake drain (Ethicon, Somerville, NJ) over the sternum, and then closed the wound in layers. The Blake drain was connected to a 10 mm flat, 3/4 fluted, and 100 cc J-VAC bulb suction reservoir for wound fluid collection. Heparin (1,000 IU) was added to the reservoir as anti-coagulant. At 24, 48, and 72 h post-surgery, the contents of the reservoir were transferred into a sterile 50 ml tube, and transported on ice for immediate analysis in the laboratory. Peripheral blood samples (10 ml) were also collected from patients before surgery, as well as at 24, 48, and 72 hours post-surgery.

**Table 1:** Demographic characteristics of patients (n = 14).

**Age (y)**	64±9
**Females**	7
**Race** 1	AA,C
**BMI**	27±4
**Cardiac surgery (Sternotomy)**	
CABG	9
Others^2^	5

1 AA, African American; C, Caucasians.

2 Other procedures involved Maze, mitral valve repair/replacements, Aortic valve repair/replacements. CABG, coronary artery bypass graft.

### Isolation of blood/wound cells and fluid

Collected wound fluid was passed through a 70 µm cell strainer to remove debris and a sample was submitted for differential cell count. Blood plasma or wound fluid (WF) was separated from wound/blood cells using centrifugation (800 g, 10 min). The plasma/fluid were stored at −80°C in small aliquots (250 µl) for further analysis. Using pelleted wound/blood cells, RBC lysis was performed with bicarbonate-buffered ammonium chloride solution (0.15 M NH4Cl, 0.01 M NaHCO3, 0.1 mM EDTA). The remaining cells were washed in phosphate buffered saline (pH 7.4) twice before proceeding with flow cytometry analysis. In some cases, cells were re-pelleted and stored frozen for protein/gene expression studies.

### Flow cytometry analysis

Peripheral blood (PB) and WF derived leukocytes (1×10^6^ cells) were stained with monoclonal FITC-conjugated anti-human CD15, CD177, CD43 (Abd Serotec, Raleigh, NC), or CD66b (BioLegend, San Diego, CA). Mouse IgG1 was used as the isotype control (Abd Serotec, Raleigh, NC). For negative control, each cell type was stained with corresponding isotype control. Cellular fluorescence was detected by exciting the fluorochrome-coated cells with a 488-nm argon laser in a C6 Accuri Flow Cytometer (Accuri Cytometers, Ann Arbor, MI) and recording FITC at 530±15 nm. For each sample, at least 10,000 gated viable cells were examined (10). Data are presented as percentage of positive cells or as mean fluorescence intensity (MFI), corrected for background fluorescence. A complete blood count (CBC) showing differential count in blood and WF samples was also obtained from The Ohio State University Central Laboratory.

### Blood plasma and wound fluid (WF) cytokine analyses

The levels of IL-4 and IL-10 were measured using commercially available enzyme-linked immunosorbent assay (ELISA) kits (all R&D Systems, Minneapolis, MN) (11). The levels of cytokines in plasma and WF were normalized against albumin concentration in the plasma/fluid. Albumin levels were determined by ELISA (AssayPro, St. Charles, MO).

### RNA isolation, reverse transcription and quantitative RT-PCR (qRT-PCR)

Total RNA was extracted using the mirVana RNA isolation kit (Ambion, Austin, TX), according to manufacturer's instructions. mRNA was quantified by real-time or quantitative (Q) PCR assay using the double-stranded DNA binding dye SYBR Green-I as described previously (12,13).

### β-Endorphin and met-Enkephalin assay

Commercially available ELISA kits were used to measure β-endorphin (MD Bioproducts, St Paul, MN) and met-enkephalin (Bachem, King of Prussia, PA) concentrations in the WF, using the manufacturer's instructions. The levels of endogenous opioids in WF were normalized to albumin levels. The intracellular levels were normalized to cellular protein content.

### HL-60 culture and differentiation

The human promyelocytic cell line HL-60 (American Type Culture Collection, Manassas, VA; ATCC code CCL-240) were cultured in RPMI 1640 with L-Glutamine, supplemented with 20% fetal bovine serum (FBS) and 1% penicillin-streptomycin (PS) (Gibco, Auckland, NZ) at 37°C and 5% CO2. To differentiate HL-60 cells into mature PMN, cells were suspended in RPMI 1640 media, supplemented with 1% PS and 20% heat-inactivated fetal bovine serum (Gibco, Auckland, NZ). Trans-retinoic acid (10 µM, Thermo Fisher, New Jersey, USA) was added to the suspension, and cells were incubated at 37°C for 5 days. The media was changed and new retinoic acid was added on day 3.

### Statistics

Data are reported as mean ± SD of at least three independent experiments. Comparisons among multiple groups were made by analysis of variance ANOVA. p<0.05 was considered statistically significant.
